# The Structure of Mediterranean Rocky Reef Ecosystems across Environmental and Human Gradients, and Conservation Implications

**DOI:** 10.1371/journal.pone.0032742

**Published:** 2012-02-29

**Authors:** Enric Sala, Enric Ballesteros, Panagiotis Dendrinos, Antonio Di Franco, Francesco Ferretti, David Foley, Simonetta Fraschetti, Alan Friedlander, Joaquim Garrabou, Harun Güçlüsoy, Paolo Guidetti, Benjamin S. Halpern, Bernat Hereu, Alexandros A. Karamanlidis, Zafer Kizilkaya, Enrique Macpherson, Luisa Mangialajo, Simone Mariani, Fiorenza Micheli, Antonio Pais, Kristin Riser, Andrew A. Rosenberg, Marta Sales, Kimberly A. Selkoe, Rick Starr, Fiona Tomas, Mikel Zabala

**Affiliations:** 1 National Geographic Society, Washington, D.C., United States of America; 2 Centre d'Estudis Avançats de Blanes, CEAB-CSIC, Blanes, Spain; 3 MOm/Hellenic Society for the Study and Protection of the Monk Seal, Athens, Greece; 4 DiSTeBA, Università del Salento, Lecce, Italy; 5 Hopkins Marine Station, Stanford University, Pacific Grove, California, United States of America; 6 NOAA Southwest Fisheries Science Center, Pacific Grove, California, United States of America; 7 Joint Institute for Marine and Atmospheric Research, University of Hawaii, Honolulu, Hawaii, United States of America; 8 U.S. Geological Survey, Hawaii Cooperative Fishery Research Unit and University of Hawaii at Manoa, Honolulu, Hawaii, United States of America; 9 Centre Mediterrani d'Investigacions Marines i Ambientals, ICM-CSIC, Barcelona, Spain; 10 Dokuz Eylül University – Institute of Marine Sciences and Technology, Inciralti, Izmir, Turkey; 11 SAD-EKOG, Maltepe, Ankara, Turkey; 12 National Center for Ecological Analysis and Synthesis, University of California Santa Barbara, Santa Barbara, California, United States of America; 13 Departament d'Ecologia, Facultat de Biologia, Universitat de Barcelona, Barcelona, Spain; 14 Université de Nice-Sophia Antipolis, Nice, France; 15 Dipartimento di Scienze Zootecniche, Università di Sassari, Sassari, Italy; 16 Scripps Institution of Oceanography, La Jolla, California, United States of America; 17 Conservation International, Arlington, Virginia, United States of America; 18 Estació d'Investigació Jaume Ferrer, IEO-Centre Oceanogràfic de Balears, Maó, Spain; 19 Moss Landing Marine Laboratories, Moss Landing, California, United States of America; Dalhousie University, Canada

## Abstract

Historical exploitation of the Mediterranean Sea and the absence of rigorous baselines makes it difficult to evaluate the current health of the marine ecosystems and the efficacy of conservation actions at the ecosystem level. Here we establish the first current baseline and gradient of ecosystem structure of nearshore rocky reefs at the Mediterranean scale. We conducted underwater surveys in 14 marine protected areas and 18 open access sites across the Mediterranean, and across a 31-fold range of fish biomass (from 3.8 to 118 g m^−2^). Our data showed remarkable variation in the structure of rocky reef ecosystems. Multivariate analysis showed three alternative community states: (1) large fish biomass and reefs dominated by non-canopy algae, (2) lower fish biomass but abundant native algal canopies and suspension feeders, and (3) low fish biomass and extensive barrens, with areas covered by turf algae. Our results suggest that the healthiest shallow rocky reef ecosystems in the Mediterranean have both large fish and algal biomass. Protection level and primary production were the only variables significantly correlated to community biomass structure. Fish biomass was significantly larger in well-enforced no-take marine reserves, but there were no significant differences between multi-use marine protected areas (which allow some fishing) and open access areas at the regional scale. The gradients reported here represent a trajectory of degradation that can be used to assess the health of any similar habitat in the Mediterranean, and to evaluate the efficacy of marine protected areas.

## Introduction

Intense exploitation over millennia has depleted Mediterranean species from the large to the small, including the Mediterranean monk seal, sea turtles, bluefin tuna, groupers, red coral, lobsters, and limpets (e.g., [1], [2], [3]). Habitat destruction, pollution, introduced species and climate change have also taken a toll on Mediterranean species and ecosystems [4], [5]. Although these impacts have been significant, based on qualitative observations over the millennia, it is difficult to evaluate their magnitude because there is no rigorous historical baseline for the abundance of marine species or the structure of marine ecosystems in the Mediterranean [6], [7], except for a few taxa and local time series of fishery dependent and independent data [3]. Most of the quantitative data on the structure of Mediterranean ecosystems originates from field studies in the last 30 years. Therefore, our attempts to evaluate the health of the marine ecosystem and the efficacy of recent conservation actions at the ecosystem level are constrained by a limited sense of what is possible or natural [8]. Here we establish the first current comparable baseline of ecosystem structure at the Mediterranean scale, focusing on nearshore rocky reefs.

What would a ‘healthy’ Mediterranean rocky bottom look like? There are no pristine sites (i.e. undisturbed by humans, with historical ecosystem structure and carrying capacity) left in the Mediterranean that allow us to set a baseline against which to compare the health of current ecosystems. Research on pristine, historically unfished sites in the central Pacific show that intact, complex reef ecosystems harbor large biomass of fishes, with inverted biomass pyramids, and high coral cover [9], [10]. Fishing pressure has been a major stressor on Mediterranean reef systems. Thus, in the Mediterranean, we would expect total fish biomass to be also the single most important indicator of the health of fish populations, with biomass increasing with decreased fishing pressure, as Mediterranean no-take marine reserves demonstrate [11], [12], [13], [14]. Therefore, marine reserves are the best proxies for the trajectory of recovery of fish assemblages towards a pristine state, possibly including cascading effects leading to a wider recovery of the protected ecosystems. However, we expect these current baselines to be still far from historical baselines with an intact ecosystem likely including all apex predators such as sharks and monk seals.

Predatory fishes can have a major role in determining the abundance of their prey and strongly modifying the ecosystem. In the Mediterranean, these effects have been observed on sea urchins, which are the major benthic herbivores on Mediterranean rocky bottoms [15], [16]. At high predatory fish abundance, predation tends to maintain low sea urchin abundances, while at low predatory fish abundance, sea urchin abundance is regulated by many other factors and thus their abundance becomes less predictable [12], [17]. The Mediterranean has only two major native herbivorous fishes, *Sarpa salpa* and *Sparisoma cretense* [18]. Although at large abundances *Sarpa salpa* should be able to reduce the biomass of some benthic algae [19], [20], only introduced herbivorous fishes (*Siganus* spp.) have been shown to cause strong algal declines (to the extent of creating barrens) in the Eastern Mediterranean [21]. The decrease of these algal communities can also affect the recruitment rate of numerous rocky fishes that select algae as settlement habitats [14], having a potential cascading effect on the whole community. We would then expect a complex, near pristine benthic community with low abundance of sea urchins and large algal biomass.

Mediterranean shallow benthic communities harbor hundreds of species of algae and invertebrates, but they tend to be dominated in cover and biomass by algae [22]. In particular, the least impacted communities are often dominated by canopies of Fucales, mostly *Cystoseira* spp. [4], [23], [24]. The abundance of *Cystoseira* appears to be determined by multiple factors, including water quality, sea urchin grazing, coastal development, and historical and current fishing pressure [25], [26], [27], [28], [29]. Fucales suffered a long-term decline in the NW Mediterranean in the last century due to a combination of the above direct and indirect human impacts [26]. Introduced algae have been present in the Mediterranean since the nineteenth century, but their number and impact on native benthic communities has increased exponentially over time [30], [31].

In addition to the direct and indirect impacts of overexploitation, there have been other major impacts to Mediterranean nearshore reefs. Historically, land use changes in the Mediterranean region had accompanying changes in nutrients and sedimentation, and a major loss of coastal habitats [4]. The Mediterranean is also increasingly affected by climate change. Seawater temperatures are steadily increasing, extreme climatic events and related disease outbreaks are becoming more frequent, faunas are shifting, and invasive species are spreading [32], [33].

There has been a great deal of work on the direct and indirect effects of human impacts on fish and benthic communities in the Western Mediterranean. However, most studies have investigated the individual effects of a number of natural and human disturbances, and processes driving observed changes remain untested across appropriately large ecosystem and geographical scales [34]. Experimental manipulation of fishing, pollution, habitat degradation, invasions and climate change impacts across the Mediterranean is not feasible. The only practical way to address the spatial distribution of community structure and its relationship with environmental and human factors is by measuring community structure across gradients of these factors.

Examples of successful recovery at the ecosystem level are rare for the Mediterranean and are systematically related to the presence of marine protected areas (MPAs) [15], [35]. This study provides the first current baseline and trajectory of degradation and recovery for Mediterranean rocky reefs, against which the present condition of existing and candidate marine protected areas can be assessed. More broadly, we provide a framework and methodology for establishing such gradients of degradation in the absence of historical data for the Mediterranean and other marine ecoregions. This Mediterranean-wide baseline should allow us to understand the magnitude of human impacts on nearshore rocky reefs, a key habitat with critical functional importance, and better evaluate the results of management and conservation actions.

## Methods

### Habitat, sampling sites and spatial replication

**Figure 1:**
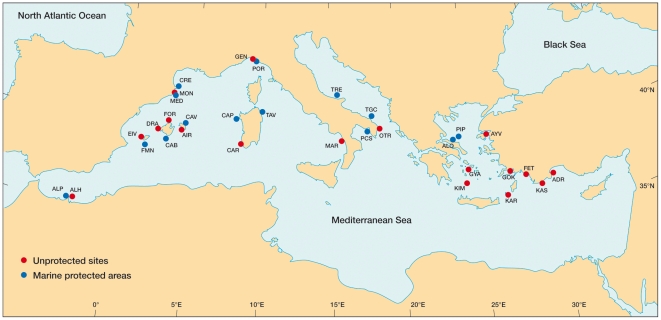
Map of study locations. ADR = Adrasan, AIR = Illa de l'Aire, ALH = Al-Hoceima (unprotected), ALO = Alonissos, ALP = Al-Hoceima MPA, AYV = Ayvalik, CAB = Cabrera, CAP = Capo Caccia, CAR = Carloforte, CAV = Cap de Cavalleria, CRE = Cap de Creus, DRA = Dragonera, EIV = Eivissa, FET = Fethiye, FMN = Formentera-Espardell, FOR = Cap Formentor, GEN = Genoa, GOK = Gökova, GYA = Gyaros, KAR = Karpathos, KAS = Kas, KIM = Kimolos-Polyaigos, MAR = Maratea, MED = Medes Islands, MON = Montgrí, OTR = Otranto, PCS = Porto Cesareo, PIP = Piperi, POR = Portofino, TAV = Tavolara, TGC = Torre Guaceto, TRE = Tremiti. For details on protection level see Figs. 2–3 and Table 1.

We conducted SCUBA surveys in May–June 2007 and 2008 at 13 MPAs and 17 unprotected areas across the Mediterranean (Spain, Italy, Greece, Turkey; Fig. 1, Table S1). One additional MPA and one fished site were surveyed in Morocco in April 2010, for a total of 32 sites. We sampled rocky habitats at 8–12 m depth, at 4–6 replicate stations within each site, according to their extension.

### Fish censuses

Fish data were collected using standard underwater visual census techniques (e.g., [9]). Sampling stations within sites were spaced at least 1 km apart from the next, except in very small marine reserves (e.g., Portofino) where we sampled stations hundreds of meters away in order to have enough replicate surveys. We conducted three replicate 25 m-long and 5 m-wide transects at each station. Along each transect, the diver swam one way at constant speed, identifying and recording the number and size of each fish encountered. Fish sizes were estimated visually in 2 cm increments of total length (TL). Fish biomass (wet mass) was estimated from size data by means of length-weight relationships from the available literature and existing databases [36], [37]. We focused on fish biomass in our analysis instead of fish density because biomass is the single most important indicator of the health of fish assemblages across gradients of human disturbance (e.g., [9], [38]). For our analysis, we assigned each fish taxon to one of four trophic groups using the information about diet in the literature [37], and in previous Mediterranean studies [12], [39]: apex predators, carnivores, herbivores and detritivores, and planktivores (Table S2).

### Benthic algae

We scraped all non-encrusting algae in five replicate 20×20 cm quadrats at each station. Each sample (quadrat) was placed in an individual zip-lock bag and brought to our field laboratory. After the erect algae were removed from a quadrat underwater, the diver estimated the cover (as % of the quadrat) of encrusting algae. After the dive, samples were stored in a cooler and transported to the field lab in seawater, without any preservative. Algal biomass was measured the same day of collection. Samples were individually sorted and weighed. Water excess from every sample was removed using a salad spinner; samples were spun for 30 seconds each. Erect algae were sorted and weighed to species on a digital balance (±0.1 g). Turf algae were identified to species in the field lab when possible, otherwise weighed as a group. Introduced species were always identified to species and weighed individually. We considered all introduced species mentioned in the scientific literature [40]. The biomass of encrusting algae was determined using the cover estimated in the field and conversion rates from the literature [41]. Algal biomass was pooled into the following groups for data analysis: (native) canopy algae (Fucales: *Cystoseira* spp. and *Sargassum* spp.), erect algae (e.g., Dictyotales), turf algae (e.g., small filamentous algae), encrusting corallines (e.g., *Lithophyllum*), encrusting non-corallines (e.g., *Lobophora*), and introduced algae.

### Benthic invertebrates

Sea urchins: we recorded sea urchin density and size (1 cm size classes, test without spines) using a plastic caliper on thirty 50 cm×50 cm quadrats per station. Biomass was then estimated using size-biomass conversion rates from the literature [42].

Sessile invertebrates: we estimated the cover of major taxa of sessile invertebrates using the point-count method on a 25 m-long transect with marks every 20 cm. We identified the organisms to the lowest possible taxonomic level. For instance, most sponges and tunicates were identified to species, although smaller colonial organisms such as bryozoans were identified as a group when identification *in situ* was not possible. We then transformed the data on area covered by benthic groups to wet mass using conversions from the literature [41]. For analysis we pooled taxa into larger functional groups (see Data Analysis).

### Environmental and human-related data

We collected habitat and environmental data for all stations *in situ*, including latitude, longitude, bottom rugosity, degree of exposure, and depth to try to minimize variability in the benthic and fish community due to habitat alone [11].

We measured the rugosity of the bottom (or surface relief) as an indicator of the structure of the physical habitat, which is known to have an important role in determining the abundance of fish (e.g., [11], [43]). To measure rugosity, a 10-m long small link chain (1.3 cm per link) was draped along the length of the centerline of each transect [44]. Care was taken to ensure that the chain followed the contour of all natural fixed surfaces directly below the transect centerline. A ratio of 10 to the linear horizontal distance between the beginning of the transect and the end of the draped chain gave an index of rugosity.

To measure the influence of the oceanographic climate we obtained 8-year averages of satellite-derived mean and median seawater surface temperature (SST) and net primary productivity (PP). SST was derived from the Pathfinder Version 5 Advanced Very High Resolution Radiometer (AVHRR) data set [45]. Vertically integrated PP was calculated by applying the method of Behrenfeld and Falkowski [46] to surface chlorophyll-a concentration, photosynthetically available radiation and sea surface temperature provided by the Moderate-resolution Imaging Spectrometer (MODIS) carried aboard NASA's Aqua spacecraft. Time series data from July 2002–June 2010 were extracted from monthly averages using 5×5 km boxes centered on each study site. Means and medians for each site were then produced from these time series.

To measure combined human impacts at the study sites we used a cumulative human impact score determined via an assessment of the cumulative impact to Mediterranean marine ecosystems resulting from multiple pressures, including fishing, climate change, pollution and biological invasions. This analysis builds on a previous global analysis of cumulative human impacts that involved combining global spatial data on 17 types of human pressures, the distribution of 20 marine ecosystem types, and scores representing the potential impact of each pressure on each ecosystem type derived through an expert judgment survey approach [47]. Impact scores determined for each pressure-ecosystem type combination are summed, within each 1 km^2^ of the ocean, to calculate the cumulative impact score. Micheli et al. (in prep.) replaced some of the data layers and included additional data to better reflect the specific pressures and ecosystems of the Mediterranean basin. A total of 22 spatial datasets of human activities and stressors and 19 ecosystem types were assembled and used in the analyses (http://globalmarine.nceas.ucsb.edu/mediterranean/). Here, we used the footprint (i.e., the cumulative impact score) of all pressures acting on a 3-km radius around each of our field sites as a measure of cumulative human impact on our focal rocky reef ecosystems. See Supporting Information S1 for more details on the cumulative human impact analysis.

The MPAs studied here include a range of protection levels (from no-take marine reserves to areas with virtually no regulations) and enforcement levels (Table 1). Based on available scientific information, personal experience and knowledge of the MPAs, and interviews with MPA staff reporting on the overall management effectiveness [48], MPAs were categorized as follows: (a) well-enforced no-take reserves (Formentera-Espardell, Medes, Portofino, Torre Guaceto, Tavolara), (b) MPAs where some fishing is allowed or some fishing occurs due to weak enforcement (Cabrera, Cap de Creus, Capo Caccia, Porto Cesareo, Cavalleria), and (c) poorly-enforced MPAs (Al-Hoceima, Alonissos, Piperi, Tremiti) and open access areas (Table 1). Some of our study sites may have been protected after our study period (e.g., Karpathos), but here we report only the level of protection at the time of our sampling. Unprotected sites are typically open-access with little enforcement of fishing regulations. To minimize differences possibly deriving from other human threats combined to fishing, sites were selected within areas not directly affected by evident sources of impact (e.g. harbors, defense structures, sewages, strong urbanization).

**Table 1:** Characteristics of the marine protected areas investigated in this study.

Name	Zone sampled	Total MPA size (ha)	Size of no-take area (ha)	Year of creation	Age of reserve at sampling (years)	Enforcement level
Al-Hoceima	Partial protection	19600	9400	2004	4	Low
Alonissos-Northern Sporades	Partial protection	207000	438	1992	16	Low
Cabrera	Partial protection	10021	360	1991	16	High
Nord de Menorca (Cavalleria)	Partial protection	5119	1100	1999	8	High
Cap de Creus	Partial protection	3056	21	1998	9	Medium
Capo Caccia	No-take	2631	38	2002	6	Medium
Formentera-Espardell	No-take	13617	400	1999	8	High
Medes Islands	No-take	94	94	1983	24	High
Piperi	No-take	438	438	1992	16	Low
Porto Cesareo	No-take	16654	173	1997	10	Low
Portofino	No-take	346	18	1998	9	High
Tavolara	No-take	15357	529	1997	11	High
Torre Guaceto	No-take	2227	179	1991	16	High
Tremiti	No-take	1466	180	1989	19	Medium

All these marine protected areas had partial protection, with areas where some types of fishing are permitted, and small no-take areas. Piperi is the no-take area of the Marine Park of Alonissos-Northern Sporades. Enforcement level *sensu* Guidetti et al. [48].

### Data analysis

Univariate analysis on total fish biomass was conducted to examine differences among sites and stations, and to test whether fish biomass responded to protection. Effects of protection were analyzed using three-way permutational analysis of variance (PERMANOVA) [49] on square root-transformed data and based on Euclidean distance dissimilarity matrix. The sampling design consisted of 3 factors: protection (fixed), site (nested in protection), and station (nested in site). For the PERMANOVA we used three levels of protection: no-take reserves, MPAs that allow some fishing, and unprotected areas. Finally, fish taxa were pooled into trophic groups (Table S2) because fishing disproportionately targets species at higher trophic levels [50], and recovery from fishing potentially includes increased abundances or biomass of high-level predators and shifts in trophic structure [51]. To investigate the relationship between fish biomass in MPAs and age of the MPA, size of the reserve, the level of protection and enforcement, and environmental variables we used multiple linear regression analysis [52].

To test for differences in biomass of algae between sites we used a non-parametric Kruskal-Wallis median test. To investigate the relationship between pairs of trophic groups, we used linear and generalized linear models and their extensions to model linear and non-linear relationships, complex trajectories, heteroscedastic data and a large inter-site variation, which we incorporated by assuming that the site effect was a random variable following a normal distribution N(0, σ^2^). Although in most cases variance of the different variables increased with density, log-transforming the variables usually stabilized the residuals and linearized the relationships. When transformation did not linearize the relationships, we assumed a Gamma distribution for the regressed variable, i.e. we assumed that the sampled value of the regressor was a draw from a random variable with mean μ and variance μ2/ν, where ν is a dispersion parameter. We also tested the presence of complex relationships between the variables by using Generalized Additive Mixed Models (GAMM). By doing so we had a certain degree of freedom in testing correlations between variables, and the opportunity to detect the shape of significant relationships.

To describe the pattern of variation in community structure (patterns of distribution of biomass/abundance of functional groups within the community) and their relationship to environmental and human gradients we used linear ordination methods. Linear models are appropriate for our data because a preliminary detrended correspondence analysis showed short gradient lengths (<2 SD) [53]. To explore the spatial distribution of community structure across the Mediterranean and its relationship with environmental variables we performed a direct gradient analysis (redundancy analysis: RDA) [54] on log-transformed data using the ordination program CANOCO for Windows version 4.0 [54], and the species and the environmental data matrices. The RDA introduces a series of explanatory (environmental) variables and resembles the model of multivariate multiple regression, allowing us to determine what linear combinations of these variables determine the gradients. We pooled data from all taxa into the following groups to facilitate the large-scale analysis: biomass of fish trophic groups, canopy algae, other algae, introduced algae, sea urchins, suspension/filter feeders, and cover of bare rock.

The environmental data matrix included the following variables: mean surface seawater temperature, mean primary productivity, bottom rugosity, protection level, and cumulative human impact. Latitude was significantly correlated to mean and median SST (Spearman rank order correlation, p<0.001, r^2^ = 0.56 and 0.48, respectively) and mean and median PP (p<0.01, r^2^ = 0.24 and 0.22, respectively), and longitude was significantly correlated to mean and median SST (p<0.001, r^2^ = 0.56 and 0.48, respectively), thus we did not use latitude and longitude in the analysis. To rank environmental variables in their importance for being associated with the structure of communities, we used a forward selection where the statistical significance of each variable was judged by a Monte-Carlo permutation test [55].

## Results

**Figure 2:**
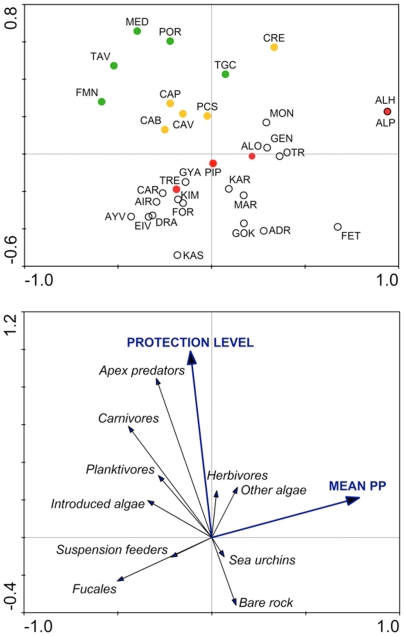
Biplot of results of redundancy analysis on biological and environmental data. Site codes as in Fig. 1. Green circles are well enforced no-take reserves, yellow circles are marine protected areas where some fishing is allowed or some fishing occurs due to weak enforcement, red circles are non-enforced MPAs, and white circles are unprotected, open access areas.

**Figure 3:**
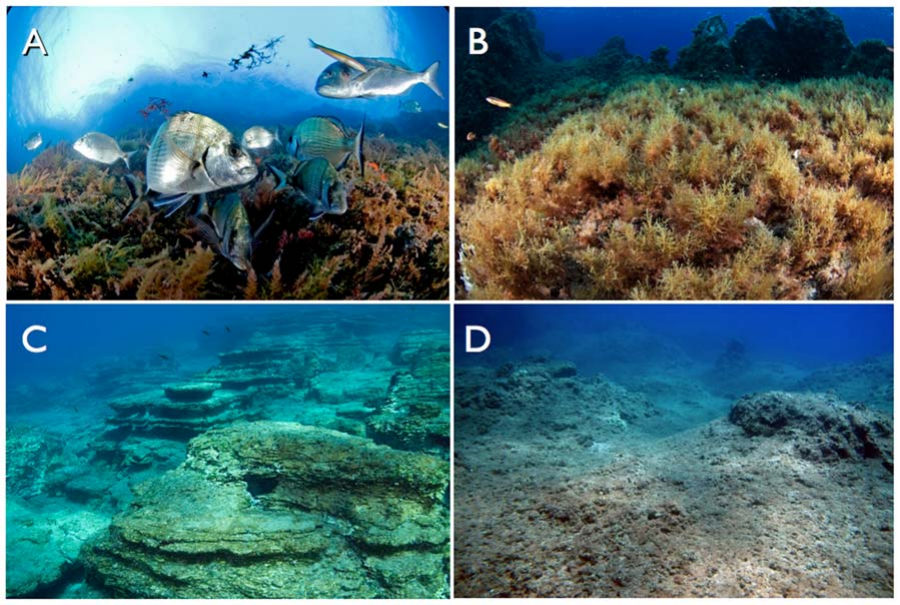
Examples of the major community states identified in this study: large fish biomass and non-canopy algae (‘predator-dominated’ ecosystems, i.e., the well-enforced marine reserves (A); ecosystems with lower fish biomass, but abundant algal canopies and suspension feeders (B); and ecosystems with lower fish biomass and extensive barrens (C), and areas covered by turf algae (D). (Photos: A,B: E Sala, C: Z Kizilkaya, D: A Vergés).

Our data reveal three groups of sites mainly constituted by: (1) well-enforced no-take marine reserves with high fish biomass, (2) partial marine protected areas and weakly enforced no-take marine reserves with lower fish biomass, and (3) non-enforced marine protected areas and areas open to fishing (Fig. 2). The main factor involved in this ordination is the protection level, which is largely correlated with the biomass of apex predators and carnivores (Figure 2). Our data also revealed a gradient along three alternative ecosystem states: (1) ecosystems characterized by large fish biomass and benthic communities dominated by non-canopy algae (‘predator-dominated’ ecosystems, i.e., the well-enforced marine reserves); (2) ecosystems with lower fish biomass, but abundant algal canopies and suspension feeders; and (3) ecosystems with lower fish biomass and extensive barrens, and areas covered by turf algae (Fig. 3).

The first two axes of the RDA biplot explained 97% of the species-environment relationship (Fig. 2, Table 2). In terms of community structure, the fit of canopy algae (Fucales) in the first axis of the RDA ordination diagram was 31%; in the second axis, the more important groups were apex predators (81%) and carnivores (55%), and bare rock (30%) in the opposite direction.

**Table 2:** Results of redundancy analysis (RDA) on log-transformed data on fish biomass, benthic biomass and cover of bare rock, and environmental and human-related variables.

Axes	1	2	3	Total variance
Eigenvalues	0.139	0.091	0.007	1.0
Species-environment correlations	0.626	0.738	0.241	
Cumulative percentage variance				
of species data	13.9	23.0	23.7	
of species-environment relation	58.7	97.0	100	
Sum of all eigenvalues				1.0
Sum of all canonical eigenvalues				0.237

Only two variables were significantly correlated to the RDA axes (Table 3). Primary production (PP) was significantly correlated with the first axis, indicating a negative relationship between PP and abundance of canopy algae and suspension feeders. PP did not have a significant relationship with the abundance of invasive species, or with the extent of sea urchin barrens (urchins and bare rock; Fig. 2). Protection level was significantly correlated with the second axis, indicating that less fishing is associated with larger fish biomass (especially with apex predators and carnivores), and more fishing is associated with less fish, more sea urchins (although weakly) and bare rock. In contrast, the abundance of canopy algae was independent of the level of protection.

**Table 3:** Conditional effects of the Monte-Carlo permutation results on the redundancy analysis (RDA).

Variable	Lambda A	F	p
Protection level	0.10	3.18	0.006
Mean PP	0.09	3.41	0.010
Human impact	0.05	1.68	0.094
Mean SST	0.04	1.55	0.178
Rugosity	0.02	1.01	0.408

PP: primary production, SST: surface seawater temperature.

Total fish biomass was significantly different among sites and stations (PERMANOVA test, p<0.001 for both factors), and ranged between 3.8 and 118 g m^−2^, a 31-fold difference (Fig. 2). Total fish biomass was significantly greater in no-take marine reserves than in MPAs that allow some fishing (p<0.001). The five highest ranking sites, in terms of fish biomass, were the well-enforced no-take reserves (Tavolara, Medes, Portofino, Torre Guaceto and Formentera-Espardell) (Fig. 2). In contrast, fish biomass in MPAs that allow some fishing were not significantly different than unprotected sites (PERMANOVA pairwise test, p = 0.16) (Fig. 2).

**Figure 4:**
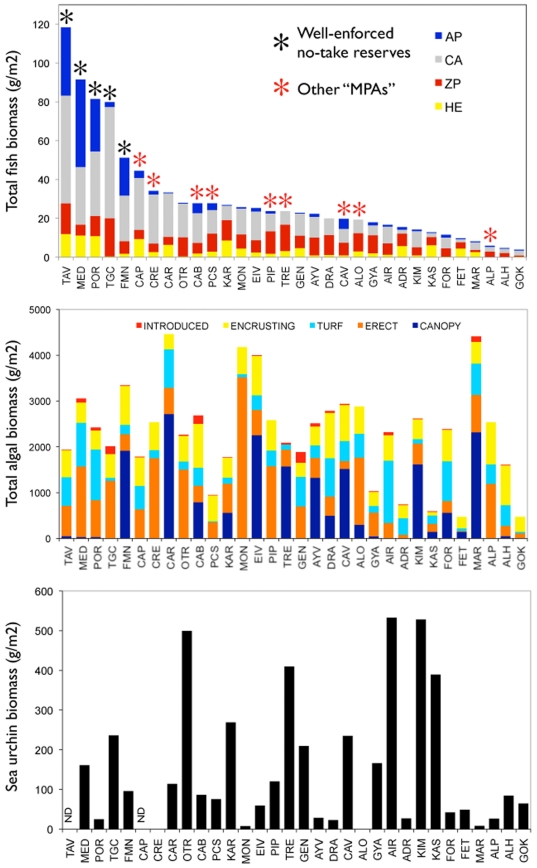
Biomass of major taxa investigated in this study. AP = apex predators, CA = carnivores, ZP = (zoo)planktivores, HE = herbivores+detritivores.

**Figure 5:**
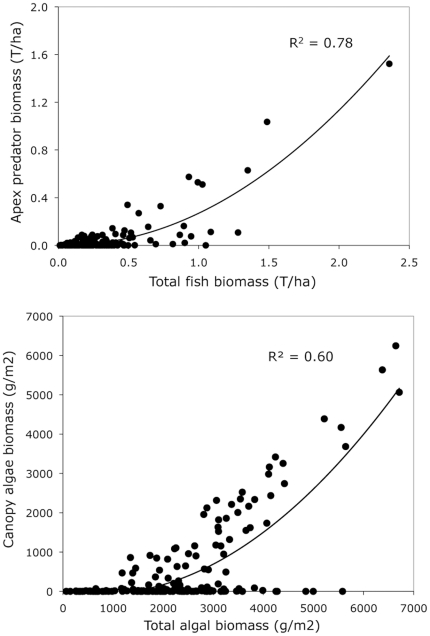
Top: Relationship between total fish biomass and biomass of apex predators. Bottom: Relationship between total biomass of algae and biomass of canopy algae (Fucales). Sites with no Fucales above 2000 g m^−2^ are found in the NW Mediterranean.

The biomass of every fish trophic group was correlated with total fish biomass (Spearman rank order correlation on sampling station averages, p<0.0001). Total fish biomass was positively related to level of protection (p = 0.038) but not to age and size of the reserve or any other variable (multiple regression, R^2^ = 0.78, p = 0.16) (Table 4). The proportion of total biomass accounted for by apex predators ranged between 0% at Tremiti and 49% at the Medes Islands Marine Reserve (Fig. 4), and the biomass of apex predators increased nonlinearly with increasing total fish biomass (R^2^ = 0.78, p<0.001) (Fig. 5). Apex predator biomass, on average, was significantly larger in protected sites than at unprotected sites (18.4% vs. 5.5%, chi-square test, p<0.001). We did not observe sharks in any of our study sites, although we observed individual monk seals (*Monachus monachus*) at Kimolos-Polyaigos, Adrasan, and Karpathos.

**Table 4:** Results of multiple linear regression on total fish biomass in the studied marine protected areas.

Variable	Partial correlation	p
Reserve size	0.01	0.986
Reserve age	0.28	0.323
Mean SST	−0.09	0.757
Mean PP	−0.06	0.926
Human impact outside reserve	−0.02	0.954
Level of protection	0.90	0.038
Bottom rugosity	0.10	0.777

The biomass of benthic algae was significantly different among sites (Kruskal Wallis median test, p<0.001) and ranged between 477 g m^−2^ at Fethiye and 4565 g m^−2^ at Carloforte, a 10-fold difference (Fig. 4). In contrast with fish biomass, there was no apparent relationship between benthic community biomass and composition and the protection status of sites. Some unprotected sites characterized by extremely low fish biomass had high algal biomass and well developed algal canopies (e.g., Maratea and Kimolos), whereas the well-enforced reserves (with the exception of Formentera-Espardell) had generally low abundance of canopy-forming algae (Fig. 4).

The relationship between total biomass of algae and biomass of canopy algae (Fucales) shows two different patterns, with some stations showing a non-linear increase after 2000 g m^−2^ of total algae, and other sites remaining at zero biomass of Fucales (Fig. 5). The latter are sites located in the NW Mediterranean (Medes, Montgrí, and Cap de Creus). For sites where Fucales are present there was a log-linear relationship between total algae and Fucales (slope = 0.00023, random effect σ = 0.545, p<0.0001).

Suspension feeders had very low biomass across sites compared to benthic algae, from 0.7 g m^−2^ to 31 g m^−2^ (for a complete list of benthic taxa encountered during the surveys see Appendix 2). The average percentage of the substrate with bare rock ranged from 0% (substrate totally covered by benthic organisms) to 31%. The sites with largest cover of bare rock were Fethiye (31%), Kas (20%), and Gökova (17%) (ANOVA, p<0.001), which harbored barrens caused by Siganidae [21].

Sea urchin biomass also differed significantly among sites (Kruskal Wallis median test, p<0.001), ranging from 0 to 533 g m^−2^ (Fig. 2). There was no significant relationship between the biomass of carnivorous fishes (or the biomass of the major predators *Diplodus* spp. [56]) and sea urchin biomass, even when accounting for the site effect and the non-normality of the data using GLMM (p-value = 0.82). Presence-absence of urchins was not related to carnivores either. Positive occurrences of urchins were not related to carnivores, protection level, and the cumulative human impact score (Supporting information S1). There was a substantial variability between sites (1.165 times the within-site variability).

There was no significant relationship between the biomass of benthic communities and total fish biomass, and between herbivorous fishes and total algal biomass at the regional scale (Supporting information S1), although in the Eastern Mediterranean, in particular in Turkey, biomass of herbivorous fishes was negatively correlated with algal biomass [21]. Unexpectedly, there was also no significant relationship between sea urchin and total algal biomass (Figure S1).

## Discussion

### Fish assemblages

Our results reveal remarkable variation in the biomass and structure of coastal ecosystems and provide the first empirical trajectory from degraded to healthy states in the Mediterranean. Such a trajectory is most clearly associated with variation in total fish biomass. We found a 31-fold range in fish biomass on rocky bottoms across the Mediterranean. If we consider the largest fish biomass values estimated in the Mediterranean at comparable habitats and depth (439 g m^−2^ [11]), the gradient of fish biomass is an extraordinary 115-fold. This is the largest fish biomass gradient ever reported for reef fish assemblages (e.g., [10], [38]), and is indicative of the large impact of historical and current intense fishing pressure in the Mediterranean. The lowest biomass values (in Gökova Bay, Turkey, and Al-Hoceima, Morocco) are among the lowest reported in the scientific literature for shallow reefs, even lower than the most overfished coral reefs in the Caribbean [38].

Apex predators are an important component of the fish assemblage by biomass at the sites with largest fish biomasses, reaching a maximum of 49% of total fish biomass at the Medes Islands Marine Reserve. This is similar to the structure found at the Papahanaumokuakea Marine National Monument in the NW Hawaiian Islands [9], and approaching the inverted biomass pyramid found at unfished reefs in the central Pacific [10], [57]. Moreover, biomass of apex predators such as the dusky grouper (*Epinephelus marginatus*) continues to increase at the Medes Island Marine Reserve after 27 years of protection [58]. These results suggest that well enforced Mediterranean marine reserves are in a trajectory of recovery similar to reserves elsewhere that moved from a degraded state with low fish biomass to biomass values similar to unfished sites (e.g., [59]).

As we expected, the highest levels of fish biomass were found only inside well-enforced no-take marine reserves. An important result of our study is that MPAs where fishing is allowed and weakly enforced no-take marine reserves had fish biomass comparable to unprotected sites. The worst performing MPA was Morocco's Al-Hoceima, which is a ‘paper park’ with virtually no management and enforcement (see [48]). Al-Hoceima was the westernmost site in our study, and it is subject to influence of Atlantic waters, which makes it the most productive of all our sites, making this result even more striking.

MPAs were located throughout the multivariate space, although they were clearly differentiated by their effective level of protection. As indicated by Guidetti & Sala [12], MPAs may have larger fish biomass than adjacent unprotected areas, but not necessarily larger biomass than unprotected areas located elsewhere. This is because of the history of fishing in each locale, local productivity, the effectiveness of MPA enforcement, and other factors that affect the overall local potential to support fish biomass. Therefore, MPAs and unprotected areas in the Mediterranean should not be regarded as two opposite situations; instead, each site should be considered within a continuum from most degraded to healthier – regardless of whether it is protected. Otherwise, the mere existence of partially protected MPAs may give the false impression that conservation is occurring.

The fish biomass levels measured in this study are not the largest in the Mediterranean (e.g., [11], [13]) although they may harbor larger biomass values following seasonal variations. Some sites, such as the Medes Islands, have significantly larger biomass in August-September when warmer waters bring piscivorous fishes to shallow waters (e.g., *Dentex dentex*, *Seriola dumerili*) (unpublished data). Therefore the ranking of fish biomass among the well-enforced marine reserves calculated in this study may exhibit some changes due to seasonal changes in fish biomass. The largest published biomass, at the Cabo de Palos Marine Reserve (439 g m^−2^ [11]) and other reserves, is restricted to very particular habitats offshore with significant water motion and currents, and probably not typical of most Mediterranean rocky reefs. These reserves have recovered successfully relative to nearby unprotected sites, and their biomass is larger than the best preserved reserves in Kenya [60], similar to the best preserved sites in the Caribbean [38], but still below the best available baselines for coral reefs in the central Pacific, where unfished islands have biomass levels up to 800 g m^−2^ [10].

Marine reserves with the largest fish biomass are a useful current baseline against which managers can compare recovery trends for fish assemblages in rocky habitats across the Mediterranean, which can be further refined by data coming from past and future studies able to identify the main drivers of recovery and degradation. It is clearly the case for the Western Mediterranean, where fish biomass achieved the highest values in the dataset (but also the lowest, at Al-Hoceima), which is consistent with documentation of successful recovery in no-take reserves from this ecoregion (e.g., [12], [13], [61]). However, there is no good estimate of the maximum potential fish biomass in the Eastern Mediterranean. Piperi, the only no-take marine reserve (as the “core area” of the National Marine Park of Alonissos, Northern Sporades) in the Aegean, has low values of fish biomass, which are comparable to unprotected areas. Piperi has dismal enforcement, and during our field surveys we observed many fishing lines tangled on the bottom as well as fishing spears stuck on rocks. Although primary productivity (PP) declines from the northwestern to the southeastern Mediterranean, our multivariate analysis showed that PP was not a significant factor in determining fish biomass across the Mediterranean – only the level of protection was. Moreover, PP values at Piperi and other Eastern Mediterranean sites are similar to the Balearic Islands, where well-enforced no-take reserves (e.g., Formentera-Espardell) have recovered fish biomass to values twice as high as Piperi. Conversely, PP is high at Al-Hoceima, Morocco, which had the lowest fish biomass recorded. In any case, because of the absence of well-enforced no-take reserves in the Southern and Eastern Mediterranean it is currently not possible to analyze the role of productivity in determining the recovery of marine reserves and their maximum biomass in the Mediterranean.

Habitat structure has been shown as an important factor in determining the structure of fish assemblages in the Mediterranean (e.g., [11]). However, we found that rugosity was not positively correlated with total fish biomass or with the structure of the whole community (fish+benthos) at the Mediterranean scale. These results indicate that the level of protection, not local habitat characteristics, is the most important factor in determining total fish biomass in the Mediterranean.

Our results are restricted to a shallow depth range (8–12 m), and comparisons with other Mediterranean locales should be restricted to similar depths. We observed larger fish biomass a little deeper in some of the study sites. For example, large dusky groupers (*Epinephelus marginatus*) were more abundant at 15–20 m depth in the Medes Islands Marine Reserve and Cabrera National Park; the goldblotch grouper *E. costae* was rarely observed in our transects in Turkey, but we commonly observed adults below 25 m depth.

### Benthic communities

Benthic communities did not follow the same gradient from healthy to degraded associated with the enforcement of protection in reserves. In fact, we did not find any effect of MPAs on benthic communities, and there was no clear pattern of the structure of benthic communities associated with the gradient in fish biomass. These results indicate that factors other than fishing are largely responsible for the structure of Mediterranean benthic communities. This is not surprising since other examples from the Mediterranean Sea demonstrated that top down mechanisms are not always the rule within MPAs, and cascading effects are likely to vary depending on local physical conditions and on the characteristics of the species that are locally dominant [62].

Unexpectedly, we did not find a strong negative correlation between the biomass of carnivorous fishes and sea urchin biomass, as it has been found for localities situated in the northwestern Mediterranean and Adriatic, where fishes above a threshold density appear to regulate sea urchin biomass [12]. The abundance of carnivorous fishes needed to exert top-down regulation on sea urchin populations is found only in well-enforced no-take reserves in the Western Mediterranean and the Adriatic Sea, even though this is not a general pattern [62], [63]. All of our Eastern Mediterranean sites had fish biomasses lower than that threshold density of predatory fishes. Because other factors come into play when predation is released, this could explain the wide variability in sea urchin biomass within and between sites. For instance, settlement of the sea urchin *Paracentrotus lividus* shows strong patchiness at scales of only tens of meters [64], [65]. Spatial and temporal variation in recruitment may mask predatory control of sea urchins, even in reserves with high predator biomass. There may also be other unknown factors such as the role of PP in the sea urchin larval pool and subsequent recruitment that could also determine the differences in the predatory fish – sea urchin relationship between the eastern and the western Mediterranean.

We would expect benthic communities with large algal biomass to be the most mature and closer to ‘pristine’. “Reference” rocky reefs in the Mediterranean with good water quality and without overgrazing by sea urchins or fish are dominated by a *Cystoseira* canopy [25], [26], [66]. *Cystoseira* biomass was also correlated with total algal biomass (except for the NW Mediterranean sites where *Cystoseira* have declined historically [26]). Therefore we expected *Cystoseira* canopies to be indicative of ‘healthy’ rocky reefs. However, most of the largest biomasses of *Cystoseira* canopies where found at unprotected sites. Among the reserves with the largest fish biomass, only Formentera-Espardell had a well developed *Cystoseira* canopy. The only example of recovery of a *Cystoseira* canopy after protection comes from the Medes Islands Marine Reserve. The Medes Islands did not have sublittoral *Cystoseira* when they were protected in 1983, but *Cystoseira* sp. became abundant after 1992 [67], [68], suggesting that recovery of formerly abundant *Cystoseira* canopies in the NW Mediterranean [26] takes longer than recovery of fish assemblages. Since dispersion of *Cystoseira* appears to be very limited, the recovery of lost canopies in large areas may prove difficult [69].

The Scandola Natural Reserve in Corsica, which we did not survey in this study, has a no-take area with large fish biomass [70] and dominant *Cystoseira* canopies [71]. Scandola is probably the best example of a ‘healthier’ rocky reef, without fishing and with good water quality, which made it one of the Mediterranean's ‘reference’ sites [25]. Nevertheless, we do not know whether recovery of *Cystoseira* at sites where it disappeared historically is facilitated by increased fish biomass within marine reserves. The recovery of fish not followed by the recovery of *Cystoseira* canopies is a signal that MPAs are embedded within large-scale human impacts impairing the effectiveness of protection and requiring management interventions following an ecosystem-based management approach.

Our redundancy analysis showed that biomass of canopy algae was negatively correlated with PP, although further analysis indicated that this may be a consequence of the NW Mediterranean having lost most of its sublittoral Fucales historically [26]. The *Cystoseira* native canopies have been disappearing throughout the Mediterranean because of direct impact by fishing nets, indirect impact by increase of water turbidity, habitat degradation, urbanization and pollution [26], [27], [29], [72]. Thus the negative correlation with PP we observed may result from concurrent loss of canopies in productive, but densely populated and developed, coastlines, not from a direct association between algal canopies and PP.

The other endpoint of benthic community structure was bare rock, which was negatively correlated to fish biomass but weakly correlated to sea urchin biomass. This result is surprising, compared to previous research in the Western Mediterranean where barrens are strongly correlated to sea urchin biomass, and may be explained by the presence of barrens in the Eastern Mediterranean caused by alien herbivorous fishes [21].

Introduced algae were not significantly related to other algae, and their biomass was on average the lowest of any algal group throughout the Mediterranean. This is an unexpected result since Mediterranean shallow water assemblages are thought to harbor the greatest number of introduced macrophytes in the world [73], with current estimates of 126 introduced macrophytes, 18 of them being considered as invasive [40]. In fact, we have detected eight of the ten top-invasive species (*Acrothamnion preissii*, *Asparagopsis armata*, *A. taxiformis*, *Caulerpa racemosa* v. *cylindracea*, *Halophila stipulacea*, *Lophocladia lallemandii*, *Stypopodium schimperi* and *Womersleyella setacea*) [30], [40] but almost none is dominant in any sample suggesting that at a Mediterranean scale and for shallow water assemblages invasive macrophytes may not be a major threat.

Our results also suggest that primary productivity (PP) may not be a limiting factor in determining the maximum biomass of benthic communities across the Mediterranean. Availability of nutrients and irradiance regulate algal biomass throughout seasonal cycles [68], [74], but there were no significant differences in average algal biomass between Western and Eastern sites, despite large differences in PP. Similarly, our overall estimates of cumulative human impact did not explain variation in algal biomass. However, results of other studies suggest that specific impacts may affect algal biomass. The major herbivores that regulate algal biomass are the sea urchins *Paracentrotus lividus* and *Arbacia lixula*, which at large densities can create extensive barrens denuding all non-encrusting algae [16], [75]. The herbivorous fish *Sarpa salpa* can also influence the biomass and vertical distribution of *Cystoseira* spp. [20]. In an experimental study on the coast of Turkey, the exclusion of the alien herbivorous fishes *Siganus luridus* and *S. rivulatus* resulted in an increase in algal biomass of up to 40 times relative to control plots within only four months [21], which shows that alien herbivorous fishes, and not PP or the cumulative human footprint, may be the major factor limiting algal growth at some Eastern Mediterranean sites. Barrens and turf algae-dominated assemblages caused by Siganidae [21] were common in our study depth range in Turkey, but below 20–25 m Siganidae were rare and erect algae were common, thus our results should be restricted to the shallower depth range we investigated. Other local impacts, e.g., from coastal development and pollution, may also contribute to degrading algal assemblages [72], even within MPAs.

### Ecological baselines

The difficulties of identifying appropriate reference conditions pose major challenges to the understanding of the causes of environmental changes [76]. There are excellent examples, however, of reconstructed historical baselines and quantitative targets for ecosystem-based management and marine conservation [3], [4], [77]. The use of historical baselines allows us to assess the history of degradation and to guide conservation and management initiatives towards new ecosystem conditions having similar structural and functional features to those of the past. However, the knowledge of “pristine conditions” (e.g., historical population level or ecosystem structure, the carrying capacity under historical ecosystem conditions) is rarely available. This reconstruction, furthermore, can be controversial in the presence of different data sources or of idiosyncratic results produced by different reconstruction methods. In the Mediterranean Sea, affected by a long history of human-induced changes and shifting baselines, the lack of reliable historical records represents a strong limit in setting meaningful reference conditions that might assist in assessing recovery. Space-for-time substitutions may represent the only solution to the lack of reliable quantitative information about historical baselines, if conclusions are derived from extensive surveys using a consistent methodology addressing multiple sites across a gradient of environmental conditions and human pressures, as in the present study. Additional snapshots of past conditions could surely help in refining management and conservation strategies.

It is important to note the absence of nearshore sharks at the study sites. Sharks and other elasmobranchs were much more abundant historically in the Mediterranean [3] and they used to be an important component of nearshore food webs [2]. The largest predators at our study sites were male dusky groupers (*Epinephelus marginatus*), which have become the dominant apex predator in biomass at most Mediterranean MPAs. Another apex predator, the Mediterranean monk seal (*Monachus monachus*), still inhabits the Aegean Sea in Greece and Turkey, and is observed often at several of our study sites (mostly at Kimolos-Polyaigos, Karpathos, Piperi, Gyaros and Adrasan) [78], [79]. Important reproductive groups have been observed at Kimolos-Polyaigos, Karpathos, Piperi and Gyaros [79], [80], [81]. It is remarkable that the monk seal, of which about 300 individuals remain in the Aegean, is more common at the sites with lowest fish biomass. The monk seal is an opportunistic predator that can swim over 60 km in a day and dive more than 100 m depth in search of prey, consuming a large variety of food sources, although the diet of the species depends to a large extend on cephalopods and particularly octopus [82]. The major factor for *M. monachus* survival appears to be the presence of suitable pupping habitat and resting caves [80], [83], which is facilitated by the presence of more than 3,000 islands and islets in the Aegean Sea, most of them uninhabited.

We did not sample the southern Mediterranean shores (except Al-Hoceima) because of the difficulties in obtaining research permits due to political issues. There are no quantitative studies on the state of the nearshore rocky reef ecosystems of the southern Mediterranean. Therefore we cannot know the community structure at the best preserved sites on the southern Mediterranean, which has the smallest number of MPAs in the Mediterranean [84].

### Applications to marine management

This study provides the first current baseline of community structure of the Mediterranean rocky sublittoral. A major insight is that, at the Mediterranean scale, partially protected MPAs (which allow some fishing) are not effective in restoring fish populations – as opposed to well enforced no-take marine reserves, which are effective. Our database can be expanded, by adding comparable data from additional sites. Managers of MPAs can place their sites on the trajectory that we have identified - from degraded (low fish biomass and bare rock) to healthier (large fish and algal biomass) - and assess the present condition of their target ecosystems relative to this trajectory. Moreover, this trajectory yields predictions regarding trends to be expected during recovery, so that, in addition to current condition, temporal change can also be interpreted to determine whether recovery is occurring. For example, monitoring a marine reserve over time and re-analyzing the data in multivariate space will allow us to determine whether and how protection is working – at the ecosystem level and across spatial scales. Thus, our empirically-derived gradient provides a practical tool for assessing ‘how your MPA is doing’ from an ecosystem perspective. Based on the current ecosystem state, management actions may be devised to promote recovery (e.g., [85]). In addition, our results provide an additional tool and criteria for guiding conservation planning and MPA site selection. Finally, evaluation of current ecosystem state in multivariate space can inform marine management, including marine spatial planning, at a larger scale in situations where multiple issues need to be addressed in order to promote recovery of rocky reef ecosystems.

## Supporting Information

Figure S1 Relationships between pairs of species groups.
(DOCX)

Table S1 List and geographic coordinates of sampling sites.
(DOCX)

Table S2 List of taxa encountered during our quantitative surveys. Fish trophic groups: AP = Apex Predators, CA = Carnivores, Pl = Planktivores, He = Herbivores.
(DOCX)

Supporting Information S1 Cumulative human impact source data and analysis.
(DOC)
