# Supplementary figures and images for: The Structure of Mediterranean Rocky Reef Ecosystems across Environmental and Human Gradients, and Conservation Implications
Source: PLoS One. 2012 Feb 29;7(2):e32742. doi: 10.1371/journal.pone.0032742 (PMC3290621; doi:10.1371/journal.pone.0032742)

**Figure S1**


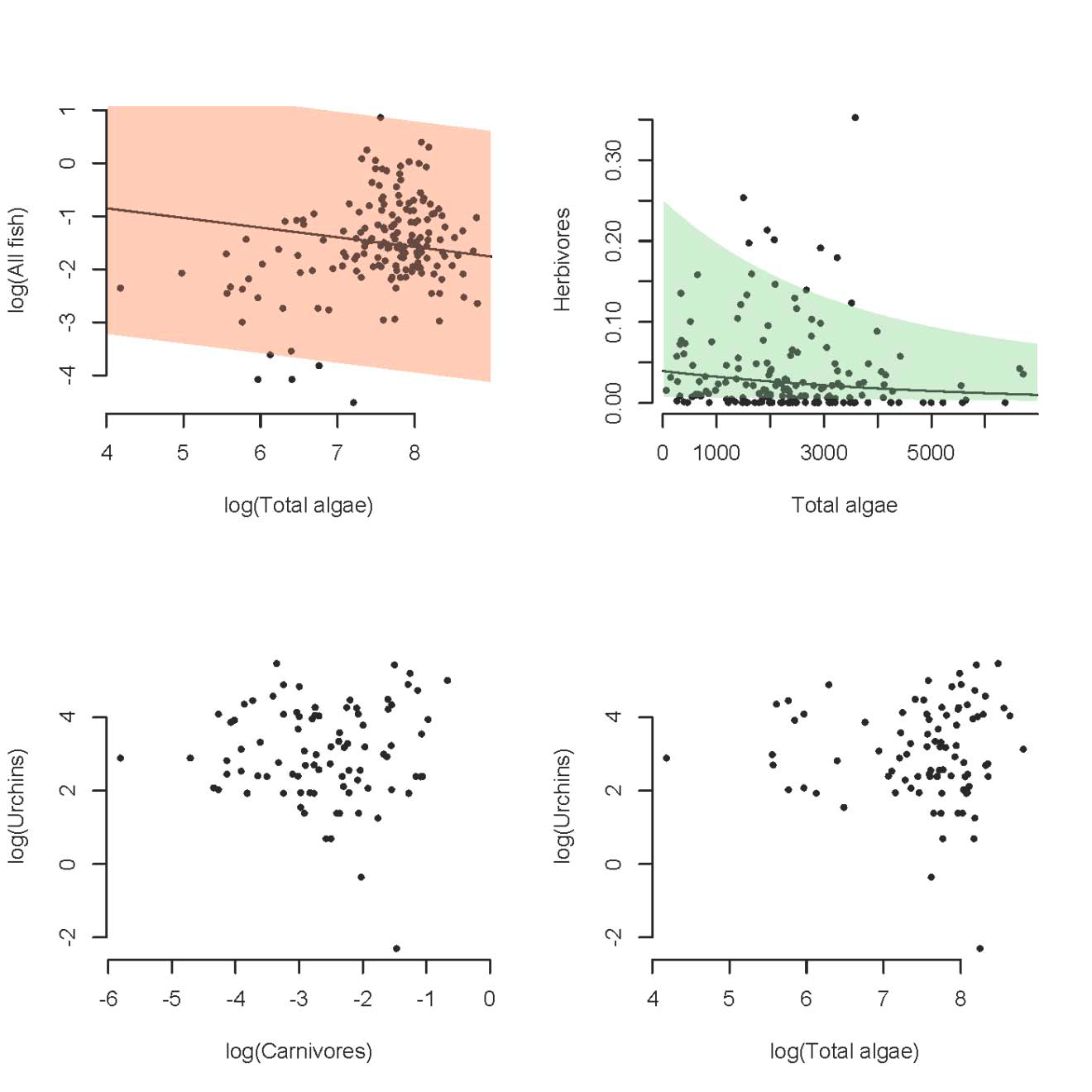

Supplement: Figure S1 — Relationships between pairs of species groups. (DOCX) [file pone.0032742.s001.docx]
