# Supplementary material for: The Structure of Mediterranean Rocky Reef Ecosystems across Environmental and Human Gradients, and Conservation Implications
Source: PLoS One. 2012 Feb 29;7(2):e32742. doi: 10.1371/journal.pone.0032742 (PMC3290621; doi:10.1371/journal.pone.0032742)
Supplement: Table S1 — List and geographic coordinates of sampling sites. (DOCX) [file pone.0032742.s002.docx]

**Table S1**

| YEAR | STATION | SITE NAME | LAT N | LONG E | DATE | MPA | No-take |
| --- | --- | --- | --- | --- | --- | --- | --- |
| 2007 | AIR1 | ILLA DE L'AIRE | 39.8031 | 4.2948 | 6-May-07 | No | N/A |
| 2007 | AIR2 | ILLA DE L'AIRE | 39.7998 | 4.2880 | 6-May-07 | No | N/A |
| 2007 | AIR3 | ILLA DE L'AIRE | 39.8215 | 4.2204 | 7-May-07 | No | N/A |
| 2007 | AIR4 | ILLA DE L'AIRE | 39.8212 | 4.2269 | 7-May-07 | No | N/A |
| 2007 | AIR5 | ILLA DE L'AIRE | 39.8009 | 4.2817 | 8-May-07 | No | N/A |
| 2007 | AIR6 | ILLA DE L'AIRE | 39.8143 | 4.2381 | 8-May-07 | No | N/A |
| 2007 | CAB1 | CABRERA | 39.1263 | 2.9578 | 18-May-07 | Yes | No |
| 2007 | CAB2 | CABRERA | 39.1380 | 2.9609 | 18-May-07 | Yes | No |
| 2007 | CAB3 | CABRERA | 39.1687 | 2.9778 | 19-May-07 | Yes | No |
| 2007 | CAB4 | CABRERA | 39.2054 | 2.9781 | 19-May-07 | Yes | No |
| 2007 | CAB5 | CABRERA | 39.1295 | 2.9294 | 20-May-07 | Yes | No |
| 2007 | CAB6 | CABRERA | 39.1326 | 2.9239 | 20-May-07 | Yes | No |
| 2007 | CAV1 | CAVALLERIA | 40.0848 | 4.0996 | 3-May-07 | No | N/A |
| 2007 | CAV2 | CAVALLERIA | 40.0849 | 4.0955 | 3-May-07 | No | N/A |
| 2007 | CAV3 | CAVALLERIA | 40.0905 | 4.0795 | 4-May-07 | No | N/A |
| 2007 | CAV4 | CAVALLERIA | 40.0741 | 4.0941 | 4-May-07 | No | N/A |
| 2007 | CAV5 | CAVALLERIA | 40.0689 | 4.0332 | 5-May-07 | No | N/A |
| 2007 | CAV6 | CAVALLERIA | 40.0585 | 4.1249 | 5-May-07 | No | N/A |
| 2007 | CRE1 | CAP DE CREUS | 42.2453 | 3.2310 | 13-Jun-07 | Yes | No |
| 2007 | CRE2 | CAP DE CREUS | 42.2471 | 3.2373 | 13-Jun-07 | Yes | No |
| 2007 | CRE3 | CAP DE CREUS | 42.3126 | 3.3105 | 14-Jun-07 | Yes | No |
| 2007 | CRE4 | CAP DE CREUS | 42.2919 | 3.3075 | 14-Jun-07 | Yes | No |
| 2007 | CRE5 | CAP DE CREUS | 42.3325 | 3.2796 | 15-Jun-07 | Yes | No |
| 2007 | CRE6 | CAP DE CREUS | 42.3235 | 3.3085 | 15-Jun-07 | Yes | No |
| 2007 | DRA1 | DRAGONERA | 39.5888 | 2.3177 | 13-May-07 | No | N/A |
| 2007 | DRA2 | DRAGONERA | 39.5985 | 2.3374 | 13-May-07 | No | N/A |
| 2007 | DRA3 | DRAGONERA | 39.5860 | 2.3274 | 15-May-07 | No | N/A |
| 2007 | DRA4 | DRAGONERA | 39.5741 | 2.3096 | 15-May-07 | No | N/A |
| 2007 | DRA5 | DRAGONERA | 39.6097 | 2.3613 | 16-May-07 | No | N/A |
| 2007 | DRA6 | DRAGONERA | 39.5632 | 2.3485 | 17-May-07 | No | N/A |
| 2007 | EIV1 | EIVISSA | 38.9787 | 1.1588 | 23-May-07 | No | N/A |
| 2007 | EIV2 | EIVISSA | 38.9690 | 1.1655 | 23-May-07 | No | N/A |
| 2007 | EIV3 | EIVISSA | 38.9563 | 1.1889 | 24-May-07 | No | N/A |
| 2007 | EIV4 | EIVISSA | 38.9598 | 1.1960 | 24-May-07 | No | N/A |
| 2007 | EIV5 | EIVISSA | 38.9879 | 1.2074 | 25-May-07 | No | N/A |
| 2007 | EIV6 | EIVISSA | 38.9885 | 1.2154 | 25-May-07 | No | N/A |
| 2007 | FMN1 | FORMENTERA | 38.7879 | 1.4806 | 26-May-07 | Yes | Yes |
| 2007 | FMN2 | FORMENTERA | 38.7988 | 1.4814 | 26-May-07 | Yes | Yes |
| 2007 | FMN3 | FORMENTERA | 38.8032 | 1.4788 | 27-May-07 | Yes | Yes |
| 2007 | FMN4 | FORMENTERA | 38.7963 | 1.4800 | 27-May-07 | Yes | Yes |
| 2007 | FMN5 | FORMENTERA | 38.7865 | 1.4848 | 28-May-07 | Yes | Yes |
| 2007 | FMN6 | FORMENTERA | 38.8043 | 1.4793 | 28-May-07 | Yes | Yes |
| 2007 | FOR1 | FORMENTOR | 39.9563 | 3.1595 | 10-May-07 | No | N/A |
| 2007 | FOR2 | FORMENTOR | 39.9460 | 3.1352 | 10-May-07 | No | N/A |
| 2007 | FOR3 | FORMENTOR | 39.9585 | 3.1715 | 11-May-07 | No | N/A |
| 2007 | FOR4 | FORMENTOR | 39.9559 | 3.1921 | 11-May-07 | No | N/A |
| 2007 | FOR5 | FORMENTOR | 39.9429 | 3.1975 | 12-May-07 | No | N/A |
| 2007 | GEN1 | GENOVA | 44.3604 | 9.1309 | 6-Jun-07 | No | N/A |
| 2007 | GEN2 | GENOVA | 44.3622 | 9.1264 | 6-Jun-07 | No | N/A |
| 2007 | GEN3 | GENOVA | 44.3708 | 9.0924 | 6-Jun-07 | No | N/A |
| 2007 | GEN4 | GENOVA | 44.3670 | 9.1065 | 13-Jun-07 | No | N/A |
| 2007 | GEN5 | GENOVA | 44.3749 | 9.0768 | 13-Jun-07 | No | N/A |
| 2007 | GEN6 | GENOVA | 44.3781 | 9.0534 | 13-Jun-07 | No | N/A |
| 2007 | MED1 | MEDES | 42.0494 | 3.2203 | 7-Jun-07 | Yes | Yes |
| 2007 | MED2 | MEDES | 42.0444 | 3.2245 | 7-Jun-07 | Yes | Yes |
| 2007 | MED3 | MEDES | 42.0414 | 3.2274 | 8-Jun-07 | Yes | Yes |
| 2007 | MED4 | MEDES | 42.0421 | 3.2253 | 8-Jun-07 | Yes | Yes |
| 2007 | MED5 | MEDES | 42.0430 | 3.2254 | 9-Jun-07 | Yes | Yes |
| 2007 | MED6 | MEDES | 42.0492 | 3.2227 | 9-Jun-07 | Yes | Yes |
| 2007 | MON1 | MONTGRI | 42.1003 | 3.1855 | 11-Jun-07 | No | N/A |
| 2007 | MON2 | MONTGRI | 42.0959 | 3.1886 | 11-Jun-07 | No | N/A |
| 2007 | MON3 | MONTGRI | 42.0873 | 3.1948 | 18-Jun-07 | No | N/A |
| 2007 | MON4 | MONTGRI | 42.0845 | 3.1971 | 18-Jun-07 | No | N/A |
| 2007 | MON5 | MONTGRI | 42.0733 | 3.2045 | 19-Jun-07 | No | N/A |
| 2007 | MON6 | MONTGRI | 42.0668 | 3.2102 | 19-Jun-07 | No | N/A |
| 2007 | OTR1 | OTRANTO | 40.1443 | 18.5067 | 15-May-07 | No | N/A |
| 2007 | OTR2 | OTRANTO | 40.1370 | 18.5156 | 15-May-07 | No | N/A |
| 2007 | OTR3 | OTRANTO | 40.1325 | 18.5168 | 15-May-07 | No | N/A |
| 2007 | OTR4 | OTRANTO | 40.1107 | 18.5181 | 25-Jun-07 | No | N/A |
| 2007 | OTR5 | OTRANTO | 40.1060 | 18.5205 | 25-Jun-07 | No | N/A |
| 2007 | OTR6 | OTRANTO | 40.0985 | 18.5099 | 25-Jun-07 | No | N/A |
| 2007 | PCS1 | PORTO CESAREO | 40.2473 | 17.8953 | 08-May-07 | No | N/A |
| 2007 | PCS2 | PORTO CESAREO | 40.2420 | 17.9015 | 09-May-07 | No | N/A |
| 2007 | PCS3 | PORTO CESAREO | 40.2369 | 17.9057 | 09-May-07 | No | N/A |
| 2007 | PCS4 | PORTO CESAREO | 40.2124 | 17.9183 | 10-May-07 | No | N/A |
| 2007 | PCS5 | PORTO CESAREO | 40.1980 | 17.9172 | 10-May-07 | No | N/A |
| 2007 | PCS6 | PORTO CESAREO | 40.1925 | 17.9182 | 10-May-07 | No | N/A |
| 2007 | POR1 | PORTOFINO | 44.3148 | 9.1576 | 5-Jun-07 | Yes | Yes |
| 2007 | POR2 | PORTOFINO | 44.3162 | 9.1615 | 14-Jun-07 | Yes | Yes |
| 2007 | POR3 | PORTOFINO | 44.3162 | 9.1650 | 14-Jun-07 | Yes | Yes |
| 2007 | POR4 | PORTOFINO | 44.3130 | 9.1657 | 5-Jun-07 | Yes | Yes |
| 2007 | TGC1 | TORRE GUACETO | 40.7225 | 17.7895 | 14-May-07 | Yes | Yes |
| 2007 | TGC2 | TORRE GUACETO | 40.7189 | 17.7959 | 14-May-07 | Yes | Yes |
| 2007 | TGC3 | TORRE GUACETO | 40.7159 | 17.8057 | 14-May-07 | Yes | Yes |
| 2007 | TGC4 | TORRE GUACETO | 40.7189 | 17.7994 | 12-May-07 | Yes | Yes |
| 2007 | TGC5 | TORRE GUACETO | 40.7137 | 17.8122 | 12-May-07 | Yes | Yes |
| 2007 | TGC6 | TORRE GUACETO | 40.7068 | 17.8266 | 12-May-07 | Yes | Yes |
| 2008 | ADR1 | ADRASAN | 36.2372 | 30.4358 | 21-May-08 | No | N/A |
| 2008 | ADR2 | ADRASAN | 36.2561 | 30.4617 | 21-May-08 | No | N/A |
| 2008 | ADR3 | ADRASAN | 36.2717 | 30.4789 | 21-May-08 | No | N/A |
| 2008 | ADR4 | ADRASAN | 36.3878 | 30.4867 | 22-May-08 | No | N/A |
| 2008 | ADR5 | ADRASAN | 36.3678 | 30.4867 | 22-May-08 | No | N/A |
| 2008 | ADR6 | ADRASAN | 36.3369 | 30.5294 | 22-May-08 | No | N/A |
| 2008 | ALO1 | ALONISSOS | 39.1082 | 23.7850 | 14-Jun-08 | No | N/A |
| 2008 | ALO2 | ALONISSOS | 39.1005 | 23.7786 | 14-Jun-08 | No | N/A |
| 2008 | ALO3 | ALONISSOS | 39.1519 | 23.8294 | 18-Jun-08 | No | N/A |
| 2008 | ALO4 | ALONISSOS | 39.1576 | 23.8407 | 18-Jun-08 | No | N/A |
| 2008 | ALO5 | ALONISSOS | 39.1719 | 23.8720 | 18-Jun-08 | No | N/A |
| 2008 | ALO6 | ALONISSOS | 39.2025 | 23.8667 | 18-Jun-08 | No | N/A |
| 2008 | AYV1 | AYVALIK | 39.3217 | 26.5455 | 4-May-08 | No | N/A |
| 2008 | AYV2 | AYVALIK | 39.3352 | 26.5928 | 4-May-08 | No | N/A |
| 2008 | AYV3 | AYVALIK | 39.3331 | 26.5683 | 5-May-08 | No | N/A |
| 2008 | AYV4 | AYVALIK | 39.3356 | 26.5627 | 5-May-08 | No | N/A |
| 2008 | AYV5 | AYVALIK | 39.3583 | 26.5900 | 6-May-08 | No | N/A |
| 2008 | AYV6 | AYVALIK | 39.3317 | 26.5303 | 6-May-08 | No | N/A |
| 2008 | CAP1 | CAPO CACCIA | 40.3615 | 8.0851 | 7-May-08 | Yes | Yes |
| 2008 | CAP2 | CAPO CACCIA | 40.3611 | 8.0835 | 7-May-08 | Yes | Yes |
| 2008 | CAP3 | CAPO CACCIA | 40.5690 | 8.2331 | 8-May-08 | Yes | Yes |
| 2008 | CAP4 | CAPO CACCIA | 40.5691 | 8.2304 | 8-May-08 | Yes | Yes |
| 2008 | CAR1 | CARLOFORTE | 39.1131 | 8.1845 | 4-May-08 | No | N/A |
| 2008 | CAR2 | CARLOFORTE | 39.1088 | 8.1600 | 4-May-08 | No | N/A |
| 2008 | CAR3 | CARLOFORTE | 39.0871 | 8.1315 | 4-May-08 | No | N/A |
| 2008 | CAR4 | CARLOFORTE | 39.0848 | 8.1380 | 5-May-08 | No | N/A |
| 2008 | CAR5 | CARLOFORTE | 39.0755 | 8.1467 | 5-May-08 | No | N/A |
| 2008 | CAR6 | CARLOFORTE | 39.0648 | 8.1487 | 5-May-08 | No | N/A |
| 2008 | FET1 | FETHIYE | 36.5572 | 29.0311 | 13-May-08 | No | N/A |
| 2008 | FET2 | FETHIYE | 36.5783 | 29.0344 | 13-May-08 | No | N/A |
| 2008 | FET3 | FETHIYE | 36.6036 | 29.0289 | 14-May-08 | No | N/A |
| 2008 | FET4 | FETHIYE | 36.6189 | 29.0417 | 14-May-08 | No | N/A |
| 2008 | FET5 | FETHIYE | 36.5961 | 29.0281 | 15-May-08 | No | N/A |
| 2008 | FET6 | FETHIYE | 36.6175 | 29.0628 | 15-May-08 | No | N/A |
| 2008 | GOK1 | GOKOVA | 37.0211 | 28.0789 | 8-May-08 | No | N/A |
| 2008 | GOK2 | GOKOVA | 37.0222 | 28.1142 | 8-May-08 | No | N/A |
| 2008 | GOK3 | GOKOVA | 37.0292 | 28.1611 | 9-May-08 | No | N/A |
| 2008 | GOK4 | GOKOVA | 37.0308 | 28.1372 | 9-May-08 | No | N/A |
| 2008 | GOK5 | GOKOVA | 37.0236 | 28.0553 | 10-May-08 | No | N/A |
| 2008 | GOK6 | GOKOVA | 37.0125 | 28.1075 | 10-May-08 | No | N/A |
| 2008 | GYA1 | GYAROS | 37.5833 | 24.7519 | 4-Jun-08 | No | N/A |
| 2008 | GYA2 | GYAROS | 37.6047 | 24.6505 | 4-Jun-08 | No | N/A |
| 2008 | GYA3 | GYAROS | 37.6001 | 24.6737 | 4-Jun-08 | No | N/A |
| 2008 | GYA4 | GYAROS | 37.6332 | 24.7387 | 6-Jun-08 | No | N/A |
| 2008 | GYA5 | GYAROS | 37.6287 | 24.7053 | 6-Jun-08 | No | N/A |
| 2008 | GYA6 | GYAROS | 37.6095 | 24.6801 | 6-Jun-08 | No | N/A |
| 2008 | KAR1 | KARPATHOS | 35.8793 | 26.8232 | 29-May-08 | No | N/A |
| 2008 | KAR2 | KARPATHOS | 35.8753 | 26.8225 | 29-May-08 | No | N/A |
| 2008 | KAR3 | KARPATHOS | 35.8685 | 27.2360 | 30-May-08 | No | N/A |
| 2008 | KAR4 | KARPATHOS | 35.8605 | 27.2385 | 30-May-08 | No | N/A |
| 2008 | KAR5 | KARPATHOS | 35.8280 | 27.2071 | 31-May-08 | No | N/A |
| 2008 | KAR6 | KARPATHOS | 35.8297 | 27.2395 | 31-May-08 | No | N/A |
| 2008 | KAS1 | KAS | 36.1508 | 29.6167 | 17-May-08 | No | N/A |
| 2008 | KAS2 | KAS | 36.1406 | 29.6617 | 17-May-08 | No | N/A |
| 2008 | KAS3 | KAS | 36.1819 | 29.6394 | 18-May-08 | No | N/A |
| 2008 | KAS4 | KAS | 36.1631 | 29.6286 | 18-May-08 | No | N/A |
| 2008 | KAS5 | KAS | 36.1875 | 29.6050 | 19-May-08 | No | N/A |
| 2008 | KAS6 | KAS | 36.1517 | 29.6300 | 19-May-08 | No | N/A |
| 2008 | KIM1 | KIMOLOS | 36.7545 | 24.6154 | 9-Jun-08 | No | N/A |
| 2008 | KIM2 | KIMOLOS | 36.7439 | 24.6338 | 9-Jun-08 | No | N/A |
| 2008 | KIM3 | KIMOLOS | 36.7475 | 24.6580 | 10-Jun-08 | No | N/A |
| 2008 | KIM4 | KIMOLOS | 36.7511 | 24.6671 | 10-Jun-08 | No | N/A |
| 2008 | KIM5 | KIMOLOS | 36.7550 | 24.5476 | 11-Jun-08 | No | N/A |
| 2008 | KIM6 | KIMOLOS | 36.7070 | 24.5473 | 11-Jun-08 | No | N/A |
| 2008 | MAR1 | MARATEA | 39.9322 | 15.7433 | 25-Jun-08 | No | N/A |
| 2008 | MAR2 | MARATEA | 39.9425 | 15.7319 | 25-Jun-08 | No | N/A |
| 2008 | MAR3 | MARATEA | 39.9908 | 15.6986 | 25-Jun-08 | No | N/A |
| 2008 | MAR4 | MARATEA | 40.0169 | 15.6739 | 26-Jun-08 | No | N/A |
| 2008 | MAR5 | MARATEA | 40.0378 | 15.6603 | 26-Jun-08 | No | N/A |
| 2008 | MAR6 | MARATEA | 40.0381 | 15.6497 | 26-Jun-08 | No | N/A |
| 2008 | PIP1 | PIPERI | 39.3326 | 24.3267 | 15-Jun-08 | Yes | Yes |
| 2008 | PIP2 | PIPERI | 39.3449 | 24.3289 | 15-Jun-08 | Yes | Yes |
| 2008 | PIP3 | PIPERI | 39.3580 | 24.3336 | 16-Jun-08 | Yes | Yes |
| 2008 | PIP4 | PIPERI | 39.3699 | 24.3306 | 16-Jun-08 | Yes | Yes |
| 2008 | PIP5 | PIPERI | 39.3681 | 24.3224 | 16-Jun-08 | Yes | Yes |
| 2008 | PIP6 | PIPERI | 39.3672 | 24.3310 | 16-Jun-08 | Yes | Yes |
| 2008 | TAV1 | TAVOLARA | 40.8761 | 9.7810 | 10-May-08 | Yes | Yes |
| 2008 | TAV2 | TAVOLARA | 40.8730 | 9.7784 | 10-May-08 | Yes | Yes |
| 2008 | TAV3 | TAVOLARA | 40.9134 | 9.7429 | 10-May-08 | Yes | Yes |
| 2008 | TAV4 | TAVOLARA | 40.9105 | 9.7380 | 11-May-08 | Yes | Yes |
| 2008 | TAV5 | TAVOLARA | 40.9021 | 9.7155 | 11-May-08 | Yes | Yes |
| 2008 | TRE1 | TREMITI | 42.2267 | 15.5439 | 30-May-08 | Yes | Yes |
| 2008 | TRE2 | TREMITI | 42.2233 | 15.7455 | 30-May-08 | Yes | Yes |
| 2008 | TRE3 | TREMITI | 42.2247 | 15.7514 | 30-May-08 | Yes | Yes |
| 2010 | ALP1 | AL-HOCEIMA MPA | 35.1399 | -4.1211 | 26-Apr-10 | Yes | No |
| 2010 | ALP2 | AL-HOCEIMA MPA | 35.2202 | -3.9815 | 26-Apr-10 | Yes | No |
| 2010 | ALP3 | AL-HOCEIMA MPA | 35.1319 | -4.1530 | 25-Apr-10 | Yes | No |
| 2010 | ALH1 | AL-HOCEIMA | 35.2392 | -3.9276 | 23-Apr-10 | No | No |
| 2010 | ALH2 | AL-HOCEIMA | 35.2373 | -3.9265 | 22-Apr-10 | No | No |
| 2010 | ALH3 | AL-HOCEIMA | 35.2392 | -3.9276 | 22-Apr-10 | No | No |
