# Supplementary material for: The Structure of Mediterranean Rocky Reef Ecosystems across Environmental and Human Gradients, and Conservation Implications
Source: PLoS One. 2012 Feb 29;7(2):e32742. doi: 10.1371/journal.pone.0032742 (PMC3290621; doi:10.1371/journal.pone.0032742)
Supplement: Table S2 — List of taxa encountered during our quantitative surveys. Fish trophic groups: AP = Apex Predators, CA = Carnivores, Pl = Planktivores, He = Herbivores. (DOCX) [file pone.0032742.s003.docx]

**Table S2**

| **ALGAE** | **ALGAL GROUP** |
| --- | --- |
| *Acetabularia acetabulum* | Turf |
| *Acinetospora crinita* | Turf |
| *Acrodiscus vidovichii* | Erect |
| *Acrosorium ciliolatum* | Turf |
| *Acrosymphyton purpuriferum* | Erect |
| *Acrothamnion preissii* | Introduced |
| *Alsidium corallinum* | Turf |
| *Amphiroa cryptarthrodia* | Erect |
| *Amphiroa rigida* | Erect |
| *Anadyomene stellata* | Turf |
| *Asparagopsis armata* | Introduced |
| *Asparagopsis taxiformis* | Introduced |
| *Asperococcus bullosus* | Turf |
| *Bonnemaisonia asparagoides* | Erect |
| *Botryocladia botryoides* | Turf |
| *Botryocladia chiajeana* | Turf |
| *Bryopsis sp.* | Turf |
| *Caulerpa prolifera* | Erect |
| *Caulerpa racemosa cylindracea* | Introduced |
| *Caulerpa racemosa lamourouxii* | Introduced |
| *Chaetomorpha* sp. | Turf |
| *Champia parvula* | Turf |
| *Chondracanthus acicularis* | Turf |
| *Chondrophycus* sp. | Turf |
| *Cladophora pellucida* | Turf |
| *Cladophora prolifera* | Turf |
| *Cladophora* sp. | Turf |
| *Cladostephus spongiosus* | Turf |
| *Codium bursa* | Erect |
| *Codium* cf. *coralloides* | Erect |
| *Codium effusum* | Erect |
| *Codium vermilara* | Erect |
| *Colpomenia sinuosa* | Erect |
| *Corallina elongata* | Turf |
| Corallinaceae articulated (species mix) | Turf |
| Corallinaceae encrusting | Encrusting corallines |
| *Cryptonemia lomation* | Erect |
| *Cutleria multifida* (*Aglaozonia* phase) | Encrusting |
| Cyanobacteria | Turf |
| *Cystoseira barbata* | Canopy |
| *Cystoseira brachycarpa balearica* | Canopy |
| *Cystoseira* cf*. elegans* | Canopy |
| *Cystoseira compressa* | Canopy |
| *Cystoseira compressa pustulata* | Canopy |
| *Cystoseira corniculata* | Canopy |
| *Cystoseira foeniculacea latiramosa* | Canopy |
| *Cystoseira foeniculacea tenuiramosa* | Canopy |
| *Cystoseira spinosa* | Canopy |
| *Dasycladus vermicularis* | Turf |
| Diatoms | Turf |
| *Dictyopteris polypodioides* | Erect |
| *Dictyota* cf. *dichotoma* | Erect |
| *Dictyota fasciola* | Erect |
| *Dictyota implexa* | Erect |
| *Digenea simplex* | Erect |
| *Dudresnaya verticillata* | Erect |
| *Elachista intermedia* | Turf |
| *Flabellia petiolata* | Erect |
| *Gelidium bipectinatum* | Turf |
| Gigartinales unidentified | Turf |
| *Halimeda tuna* | Erect |
| *Haliptilon virgatum* | Turf |
| *Halopteris filicina* | Erect |
| *Halopteris scoparia* | Erect |
| *Hildenbrandia crouaniorum* | Encrusting |
| *Hydroclathrus clathratus* | Erect |
| *Irvinea boergesenii* | Turf |
| *Laurencia* gr. *obtusa* | Turf |
| *Laurencia* sp*.* | Turf |
| *Liagora viscida* | Erect |
| *Lithophyllum* sp*.* | Encrusting corallines |
| *Lithophyllum stictaeforme* | Encrusting corallines |
| *Lobophora variegata* | Encrusting |
| *Lophocladia lallemandii* | Introduced |
| *Mesophyllum alternans* | Encrusting corallines |
| *Nereia filiformis* | Erect |
| *Padina pavonica* | Erect |
| *Palmophyllum crassum* | Encrusting |
| *Parvocaulis parvulus* | Turf |
| *Peyssonnelia harveyana* | Erect |
| *Peyssonnelia rosa-marina* | Encrusting |
| *Peyssonnelia squamaria* | Erect |
| *Polystrata fosliei* | Encrusting |
| *Pseudochlorodesmis furcellata* | Turf |
| *Pseudolithoderma adriaticum* | Encrusting |
| *Rhodymenia ardissonei* | Turf |
| *Rytiphlaea tinctoria* | Erect |
| *Sargassum vulgare* | Canopy |
| *Scinaia furcellata* | Erect |
| *Scinaia* sp*.* | Erect |
| *Sebdenia* sp*.* | Erect |
| *Sphacelaria cirrosa* | Turf |
| *Sphaerococcus coronopifolius* | Erect |
| *Stilophora tenella* | Erect |
| *Stypopodium schimperi* | Introduced |
| *Taonia atomaria* | Erect |
| *Tricleocarpa fragilis* | Erect |
| *Ulva rigida* | Erect |
| *Valonia utricularis* | Turf |
| *Womersleyella setacea* | Introduced |
| *Wrangelia penicillata* | Turf |
| *Zanardinia typus* | Erect |
| **SEAGRASSES** |  |
| *Halophila stipulacea* |  |
| *Posidonia oceanica* |  |
| Dead *Posidonia* matte |  |
| **SPONGES** |  |
| *Agelas oroides* |  |
| *Aplysina aerophoba* |  |
| *Cacospongia* sp. |  |
| Calcispongia (unidentified) |  |
| *Chondrilla nucula* |  |
| *Chondrosia reniformis* |  |
| *Cliona celata* |  |
| *Cliona* spp. |  |
| *Cliona viridis* |  |
| *Crambe crambe* |  |
| *Dysidea avara* |  |
| *Hemimycale columella* |  |
| *Ircinia dendroides* |  |
| *Ircinia fasciculata* |  |
| *Ircinia foetida* |  |
| *Ircinia spinosa* |  |
| *Ircinia* spp. |  |
| *Ircinia variabilis* |  |
| *Petrosia ficiformis* |  |
| *Phorbas fictitius* |  |
| *Phorbas* spp. |  |
| *Phorbas tenacior* |  |
| *Raspaciona aculeata* |  |
| *Sarcotracus spinosulus* |  |
| *Spongia officinalis* |  |
| **ANTHOZOANS** |  |
| *Aiptasia mutabilis* |  |
| *Anemonia sulcata* |  |
| *Balanophyllia europea* |  |
| *Caryophyllia* sp. |  |
| *Cerianthus membranaceus* |  |
| *Cladocora caespitosa* |  |
| *Cornularia cornucopiae* |  |
| *Eunicella cavolinii* |  |
| *Eunicella singularis* |  |
| *Oculina patagonica* |  |
| *Parazoanthus axinellae* |  |
| **HYDROZOANS** |  |
| *Aglaopheniidae* |  |
| *Eudendrium* spp. |  |
| *Plumularia* sp. |  |
| *Plumulariidae* |  |
| **FORAMINIFERA** |  |
| *Miniacina miniacea* |  |
| **SABELLID POLYCHAETES** |  |
| Sabellidae (unidentified) |  |
| *Sabella spallanzani* |  |
| **SERPULID POLYCHAETES** |  |
| *Salmacina dysteri* |  |
| *Serpula vermicularis* |  |
| **BIVALVES** |  |
| *Arca noae* |  |
| *Gastrochaena dubia* |  |
| *Ostrea* sp. |  |
| *Pinna nobilis* |  |
| *Spondylus gaederopus* |  |
| *Spondylus* sp. |  |
| **GASTROPODS** |  |
| *Hexaplex trunculus*  *Serpulorbis arenarius* |  |
| **NUDIBRANCHS** |  |
| *Hypselodoris orsinii* |  |
| **CEPHALOPODS** |  |
| *Octopus vulgaris* |  |
| **CRUSTACEANS** |  |
| *Balanus perforatus* |  |
| *Balanus* sp. |  |
| *Brachyura* |  |
| *Conchoderma virgatum* |  |
| **BRYOZOANS** |  |
| *Margaretta cereoides* |  |
| *Myriapora truncata* |  |
| *Reptadeonella violacea* |  |
| *Schizobrachiella sanguinea* |  |
| *Schizobrachiella* sp. |  |
| *Schizoporella dunkeri* |  |
| **ECHINODERMS** |  |
| *Arbacia lixula* |  |
| *Echinaster sepositus* |  |
| *Echinus* spp. |  |
| *Holothuria* spp. |  |
| *Paracentrotus lividus* |  |
| *Sphaerechinus granularis* |  |
| **ECHIURIDS** |  |
| *Bonellia viridis* |  |
| **TUNICATES** |  |
| *Aplydium* spp. |  |
| *Clavelina lepadiformis* |  |
| *Cystodites dellechiajei* |  |
| Didemnideae |  |
| *Halocynthia papillosa* |  |
| *Microcosmus* sp. |  |
| *Pseudodistoma crucigaster* |  |
| *Pycnoclavella* spp. |  |
| **FISHES** | **Trophic group** |
| *Anthias anthias* | Pl |
| *Apogon imberbis* | Pl |
| *Atherina hepsetus* | Pl |
| *Balistes carolinensis* | Ca |
| *Boops boops* | Pl |
| *Chelon labrosus* | De |
| *Chromis chromis* | Pl |
| *Clupea harengus* | Pl |
| Clupeidae | Pl |
| *Conger conger* | Ap |
| *Coris julis* | Ca |
| *Ctenolabrus rupestris* | Ca |
| *Dasyatis pastinaca* | Ca |
| *Dasyatis* sp. | Ca |
| *Dentex dentex* | Ap |
| *Dicentrarchus labrax* | Ap |
| *Diplodus annularis* | Ca |
| *Diplodus cervinus* | Ca |
| *Diplodus puntazzo* | Ca |
| *Diplodus sargus* | Ca |
| *Diplodus vulgaris* | Ca |
| *Epinephelus caninus* | Ap |
| *Epinephelus costae* | Ap |
| *Epinephelus marginatus* | Ap |
| *Fistularia commersonii* | Ap |
| *Gammogobius steinitzi* | Ca |
| *Gobius auratus* | Ca |
| *Gobius buchichi* | Ca |
| *Gobius cobitis* | Ca |
| *Gobius cruentatus* | Ca |
| *Gobius geniporus* | Ca |
| *Gobius paganellus* | Ca |
| *Gobius vittatus* | Ca |
| *Gobius xanthocephalus* | Ca |
| *Gobius* spp. | Ca |
| *Labrus merula* | Ca |
| *Labrus mixtus* | Ca |
| *Labrus viridis* | Ca |
| *Lichia amia* | Ap |
| *Lithognathus mormyrus* | Ca |
| *Liza aurata* | De |
| *Mugil cephalus* | De |
| Mugilidae | De |
| *Mullus barbatus* | Ca |
| *Mullus surmuletus* | Ca |
| *Muraena helena* | Ap |
| *Mycteroperca rubra* | Ap |
| *Oblada melanura* | Pl |
| *Pagellus acarne* | Ca |
| *Pagrus pagrus* | Ca |
| *Parablennius gattorugine* | Ca |
| *Parablennius incognitus* | Ca |
| *Parablennius pilicornis* | Ca |
| *Parablennius rouxi* | Ca |
| *Parablennius zvonimiri* | Ca |
| *Phycis phycis* | Ca |
| *Pomadasys incisus* | Ca |
| *Pomatoschistus quagga* | Ca |
| *Pomatomus saltatrix* | Ap |
| *Pteragogus pelycus* | Ca |
| *Sargocentron rubrum* | Ca |
| *Sarpa salpa* | He |
| *Sarda sarda* | Ap |
| *Scianea umbra* | Ca |
| *Scorpaena maderensis* | Ca |
| *Scorpaena notata* | Ca |
| *Scorpaena porcus* | Ca |
| *Scorpaena scrofa* | Ap |
| *Serranus cabrilla* | Ca |
| *Seriola dumerili* | Ap |
| *Serranus hepatus* | Ca |
| *Serranus scriba* | Ca |
| *Siganus luridus* | He |
| *Siganus rivulatus* | He |
| *Sparus aurata* | Ca |
| *Sparisoma cretense* | He |
| *Sphyraena viridensis* | Ap |
| *Spicara maena* | Pl |
| *Spicara smaris* | Pl |
| *Spondyliosoma cantharus* | Ca |
| *Stephanolepis diaspros* | Ca |
| *Symphodus cinereus* | Ca |
| *Symphodus doderleini* | Ca |
| *Symphodus mediterraneus* | Ca |
| *Symphodus melanocercus* | Ca |
| *Symphodus ocellatus* | Ca |
| *Symphodus roissali* | Ca |
| *Symphodus rostratus* | Ca |
| *Symphodus tinca* | Ca |
| *Synodus saurus* | Ap |
| *Syngnathus typhle* | Ca |
| *Thalassoma pavo* | Ca |
| *Thorogobius ephippiatus* | Ca |
| *Torquigener flavimaculosus* | Ca |
| *Torpedo torpedo* | Ca |
| *Tripterygion delaisi* | Ca |
| *Tripterygion melanurus* | Ca |
| *Trisopterus minutus* | Ca |
| *Tripterygion tripteronotus* | Ca |
