# Supplementary material for: The Structure of Mediterranean Rocky Reef Ecosystems across Environmental and Human Gradients, and Conservation Implications
Source: PLoS One. 2012 Feb 29;7(2):e32742. doi: 10.1371/journal.pone.0032742 (PMC3290621; doi:10.1371/journal.pone.0032742)
Supplement: Supporting Information S1 — Cumulative human impact source data and analysis. (DOC) [file pone.0032742.s004.doc]

**Supporting Information S1**

**General Approach**

This analysis builds on a previous global analysis of cumulative human impacts (Halpern et al. 2008) that involved combining global spatial data on 17 types of human pressures, the distribution of 20 marine ecosystem types, and scores representing the potential impact of each pressure on each ecosystem type derived through an expert judgment survey approach (Halpern et al. 2007). A detailed description of methods and criteria for selection of specific pressures is provided in these articles and associated SOM. As in Halpern et al. (2008) and additional studies (Halpern et al. 2009, Selkoe et al. 2009), we used a cumulative impact model that follows a 4-step process. We first assembled the spatial data for each anthropogenic driver (*D_i_*) and each ecosystem (*E_j_*). Second, we log[X+1]-transformed and rescaled between 0-1 each driver layer to put them on a single, unitless scale that allows direct comparison, and converted ecosystem data into 1 km^2^ presence/absence layers. Third, for each 1 km^2^ cell of ocean we multiplied each driver layer with each ecosystem layer to create driver-by-ecosystem combinations, and then multiplied these combinations by the appropriate weighting variable (*u_ij_*). These weighting variables come from an expert survey that assessed the vulnerability of each ecosystem to each driver on the basis of 5 ecological trait (Halpern et al. 2007). The weighting values represent the relative impact of an anthropogenic driver on an ecosystem within a given cell when both exist in that cell, and do not represent the relative global impact of a driver or the overall status of an ecosystem. The sum of these weighted driver-by-ecosystem combinations then represents the relative cumulative impact of human activities on all ecosystems in a particular 1 km^2^ cell. Because in these analyses our focus are rocky reef ecosystems, we limited our calculations of cumulative impact scores to this ecosystem type.

In the present Mediterranean analysis, we also replaced some of the data layers and included additional data to better reflect the specific pressures and ecosystems of the Mediterranean basin. A total of 18 spatial datasets of human activities and stressors were assembled and used in the analyses and maps (see table below).

| **Activity or stressor** | **Extent** | **Resolution** | **Source** |  |
| --- | --- | --- | --- | --- |
| Artisanal fishing | Med | 0.5 degree | GlobalMarine | |
| Benthic structures (oil rigs) | Med | 1km | GlobalMarine | |
| Coastal population density | Med | 1km | GlobalMarine | |
| Commercial shipping | Med | points | GlobalMarine | |
| Fishing (demersal, destructive) | Med | 1.0 degree | GlobalMarine | |
| Fishing (demersal, non-destructive, high bycatch) | Med | 0.5 degree | GlobalMarine | |
| Fishing (demersal, non-destructive, low bycatch) | Med | 0.5 degree | GlobalMarine | |
| Fishing (pelagic, high by-catch) | Med | 0.5 degree | GlobalMarine | |
| Fishing (pelagic, low by-catch) | Med | 0.5 degree | GlobalMarine | |
| Invasive species | Med | 1km | CIESM |  |
| Nutrient input (fertilizers) | Med | 1km | GlobalMarine | |
| Ocean acidification | Med | 1.0 degree | GlobalMarine | |
| Oil spills | Med | 1km | REMPEC |  |
| Organic pollution (pesticides) | Med | 1km | GlobalMarine | |
| Risk of hypoxia | Med | 1.5km | EEA JRC |  |
| Sea Surface Temperature change | Med | 4km | EEA JRC |  |
| Urban runoff (nonpoint inorganic pollution) | Med | 1km | GlobalMarine | |
| UV radiation | Med | 1km | GlobalMarine | |

Maps of individual data layers can be viewed at: <http://globalmarine.nceas.ucsb.edu/mediterranean/>

Data used in Halpern et al. (2008) are described in detail in the SOM of that paper. Below we describe the additional datasets.

*Invasive species*

Halpern et al. (2008) modeled the incidence of invasive species as a function of the amount of cargo traffic at a port, on the basis of results from other studies showing a relationship between these two variables and in the absence of actual data for the global distribution of invasive species. In this analysis, we replaced this layer with data on the actual distribution of a subset of invasive species in the Mediterranean. We used maps compiled by The Mediterranean Science Commission (CIESM Atlas of Exotic Species, [www.ciesm.org](http://www.ciesm.org/)) on the distribution of exotic crustaceans, fish, molluscs and macroalgae in the Mediterranean. Based on a review of the literature and consultation with experts, we selected a subset of 20 harmful invasives with documented ecological impacts, then overlayed their distribution to produce a datalayer and map representing the number of harmful marine invasives reported within each 1 km^2^ of intertidal and shallow subtidal (down to 50 m depths) coastal habitat.

*Oil spills*

We obtained data on the occurrence and magnitude of ship accidents resulting in oil spills between 1977-2009, compiled by the Regional Marine Pollution Emergency Response Centre for Mediterranean Sea (REMPEC).

*Climate change (SST increase)*

We used estimates of rates of change in sea surface temperature (SST) as a measure of pressure from temperature warming. The detailed spatial analysis of rates of change in sea surface temperature, utilizing remote sensing data for 1985-2006, was performed by L. Nykjaer, from the Joint Research Center (JRC) of the European Environmental Agency (EEA) (Nykjaer 2009). This data layer replaced calculations of the frequency of SST anomalies used in the global analysis.

*Risk of hypoxia*

The semienclosed setting of the Mediterranean and the high coastal population density make coastal areas of the Mediterranean Sea prone to anthropogenic eutrophication and associated formation of hypoxic and anoxic waters. To capture this important pressure on Mediterraenan coastal marine ecosystems, we included in our analysis and additional data layers representing the risk of development of hypoxic conditions. The risk of development of low-oxygen conditions was predicted based on a biophysical model (OxyRisk) developed by Djavidnia et al. (2005) at the Joint Research Center (JRC) of the European Environmental Agency (EEA). We used the modeled output to identify areas susceptible to hypoxia.

REFERENCES

Djavidnia, S. et al. 2005. Oxygen Depletion Risk Indices - OXYRISK & PSA V2.0: New developments, structure and software content. *JRC Report*.

Halpern, B. S., K. A. Selkoe, F. Micheli, C. V. Kappel. 2007. Evaluating and ranking the vulnerability of marine ecosystems to anthropogenic threats. *Conservation Biology* 21: 1301-1315.

Halpern, B.S., S. Waldbridge, K.A. Selkoe, C. V. Kappel, F. Micheli and 14 others. 2008. A global map of human impact on marine ecosystems. *Science* 319: 948-952.

Nykjaer, L. 2009. Mediterranean Sea surface warming 1985–2006. *Climate Research* 39: 11-17.
